# Influence of nonalcoholic fatty liver disease severity on carotid adventitial vasa vasorum

**DOI:** 10.3389/fendo.2024.1366015

**Published:** 2024-05-07

**Authors:** Josep León-Mengíbar, Enric Sánchez, Ferrán Herrerías, Mari Cruz De La Fuente, Maite Santamaría, José Manuel Valdivielso, Marcelino Bermúdez-López, Eva Castro, Judit Pallarés, Xavier Matias-Guiu, Felip Vilardell, Assumpta Caixàs, Marta Bueno, Raquel Martí, Albert Lecube

**Affiliations:** ^1^ Endocrinology and Nutrition Department, University Hospital Arnau de Vilanova, Lleida, Spain; ^2^ Obesity, Diabetes and Metabolism (ODIM) Research Group, Institut de Recerca Biomèdica de Lleida (IRBLleida), Lleida, Spain; ^3^ Medicine and Surgery Department, University of Lleida, Lleida, Spain; ^4^ Gastrointestinal Surgery Department, Arnau de Vilanova University Hospital, Lleida, Spain; ^5^ Surgery Research Group, Institut de Recerca Biomèdica de Lleida (IRBLleida), Lleida, Spain; ^6^ Vascular and Renal Translational Research Group, Institut de Recerca Biomèdica de Lleida (RBLleida), Lleida, Spain; ^7^ Department of Pathology and Molecular Genetics, Arnau de Vilanova University Hospital, Institut de Recerca Biomèdica (IRB) and University of Lleida, Lleida, Spain; ^8^ Endocrinology and Nutrition Department, Parc Taulí Hospital Universitari, Institut d’Investigació i Innovació Parc Taulí (IPT-CERCA), Medicine Department, Universitat Autònoma de Barcelona, Sabadell, Spain

**Keywords:** atherosclerosis, vasa vasorum, vascular disease, nonalcoholic fatty liver disease, obesity

## Abstract

**Introduction:**

Nonalcoholic fatty liver disease (NAFLD) affects a quarter of the world’s population and encompasses a spectrum of liver conditions, from non-alcoholic steatohepatitis (NASH) to inflammation and fibrosis. In addition, NAFLD also links to extrahepatic conditions like diabetes or obesity. However, it remains unclear if NAFLD independently correlates with the onset and progression of atherosclerosis.

**Material and methods:**

This cross-sectional study aimed to explore the relationship between NAFLD severity, assessed via liver biopsy, and early atherosclerosis using adventitial vasa vasorum (VV) density. It included 44 patients with obesity (33 with steatosis, 11 with NASH) undergoing bariatric surgery.

**Results:**

Results revealed no significant differences in adventitial VV density between steatosis and NASH groups, neither in the mean values [0.759 ± 0.104 vs. 0.780 ± 0.043, P=0.702] nor left-right sides. Similarly, carotid intima-media thickness (cIMT) did not vary between these groups. Additionally, no linear correlation existed between VV density and cIMT. Only gender showed an association with VV density.

**Conclusion:**

These findings suggest that NASH severity doesn’t independently drive early atherosclerosis or affects cIMT. Gender might play a role in early atherosclerotic disease in NAFLD, impacting VV density and cIMT. This highlights the need to consider other risk factors when evaluating cardiovascular risk in NAFLD patients.

## Introduction

Nonalcoholic fatty liver disease (NAFLD) is a highly prevalent and potentially life-threatening illness that has become the world’s most common chronic liver disease, as well as the second most common indication for liver transplant (1, 2). It affects around a quarter of the world’s population (95% CI 22.1-28.6), and this prevalence continues to increase globally, exacerbated by the obesity epidemic (3, 4). NAFLD is a heterogeneous disease that encompasses a spectrum of conditions, ranging from simple steatosis to cirrhosis and hepatocellular carcinoma with an intermediate stage known as non-alcoholic steatohepatitis (NASH) (5). NASH is characterized by inflammation, fibrosis and the onset of structural changes in the liver (6, 7). In addition to its impact on liver health, NAFLD is strongly associated with extrahepatic diseases such as obesity, insulin resistance, type 2 diabetes mellitus, hypertension, atherogenic dyslipidemia, and alterations in the gut microbiome (8). Furthermore, certain studies have demonstrated the involvement of oxidative stress, procoagulant factors, and systemic inflammation mediators, including interleukin 6, tumor necrosis factor α, and C-reactive protein, in NAFLD pathogenesis (9, 10).

It has been proposed that the presence of NAFLD also contributes to vascular inflammation and tone, thereby promoting the development of endothelial dysfunction and atherosclerotic plaques, ultimately heightening the risk of cardiovascular (CV) disease (9–11). However, a consensus regarding whether NAFLD is independently associated with an increased CV risk in the absence of other established risk factors is lacking (12). Furthermore, its correlation with surrogate markers of CV disease, such as carotid intima-media thickness (cIMT), aortic stiffness, brachial artery vasodilatory responsiveness, and coronary artery calcification, remains to be fully elucidated (9, 10, 13, 14).

While cIMT currently stands as one of the most widely employed predictors of atheromatosis (15), a growing body of evidence substantiates the notion that the atheromatous process initiates earlier, involving hyperplasia and pathological expansion of the adventitial vasa vasorum (VV) into the avascular intima (16). Consequently, the visualization of adventitial VV holds the potential to detect atherosclerosis development well in advance of any discernible increase in cIMT, thereby facilitating the early identification of individuals at elevated risk of CV disease. Despite this, the impact of NAFLD on VV structure and function remains unexplored.

Thus, the objective of our study was to assess whether the severity of NAFLD, diagnosed via liver biopsy, correlates with the onset and progression of atherosclerosis by examining the VV. To achieve this, we conducted a single-center cross-sectional study involving 44 patients with severe obesity (33 patients with simple hepatic steatosis and 11 patients with NASH) who subsequently underwent bariatric surgery.

## Materials and methods

### Ethical considerations

The research conducted in this study received approval from the Human Ethics Committee at Arnau de Vilanova University Hospital (CEIC-1275). The details of the study protocol were provided to all patients scheduled for a bariatric procedure in 2017, and they were extended an invitation to take part. Those patients who chose to participate provided their informed consent through a signed written document. The study adheres to the ethical principles outlined in the 1975 Declaration of Helsinki. Importantly, this was an observational study, and as such, it did not necessitate registration as a clinical trial.

### Study population

We assessed the density of adventitial VV based on the presence of NAFLD in a single-center analytical cross-sectional study involving 33 individuals with simple steatosis and 11 cases with NASH. The study was conducted at the Arnau de Vilanova University Hospital from Lleida (Lleida, Spain). Using the standard deviation (SD) of adventitial VV determined from a previous investigation, we calculated that a minimum sample size of 35 subjects was required (17). Thus, we contacted all 73 individuals scheduled for a programmed bariatric procedure in 2017 and invited them to participate in an outpatient clinic visit before the procedure.

**Figure 1:**
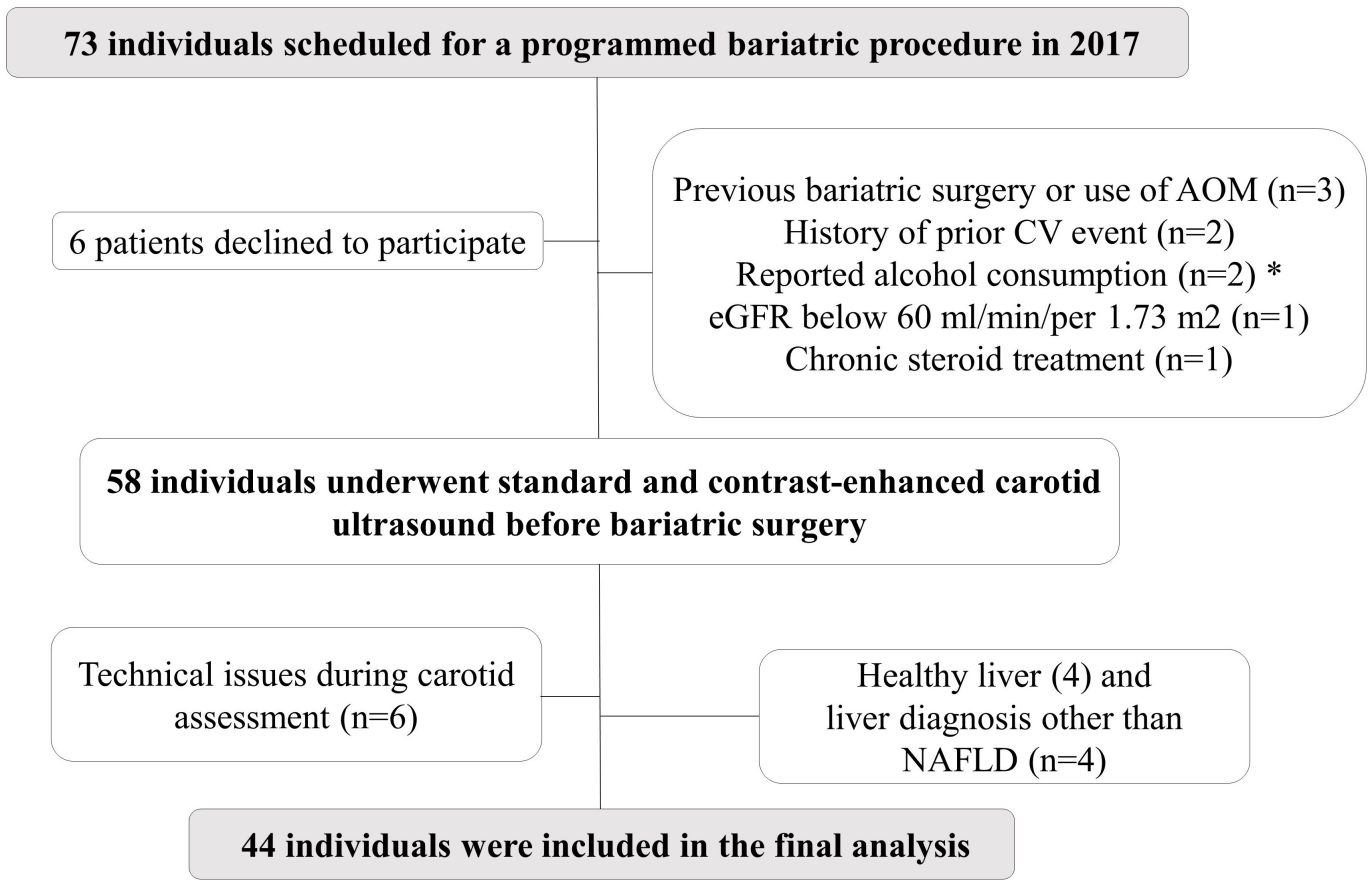
Flowchart of the study population. AOM, anti-obesity medications; CV, cardiovascular; eGFR, estimated glomerular filtration rate; NAFLD, nonalcoholic fatty liver disease. *: ≥20g/day in women or 30mg/day in men.

While all 73 patients met the eligibility criteria for gastrointestinal surgery as outlined in the National Institutes of Health Consensus Conference guidelines (18), we excluded nine individuals for various reasons: previous bariatric surgery or use of anti-obesity medication (n=3), history of prior cardiovascular event (n=2), reported alcohol consumption ≥20g/day in women or 30mg/day in men (n=2), glomerular filtration rate below 60 ml/min/per 1.73 m^2^ (n=1), and chronic steroid treatment (n=1). See the flowchart of the study in **Figure 1**. Additionally, 6 patients declined to participate, and 6 patients were excluded from the final analysis due to technical issues (rapid contrast clearance impeding proper VV density assessment or missing reports of VV or cIMT).

Upon availability of liver biopsy data, 4 patients were excluded for having a healthy liver and 4 patients were excluded due to hepatic conditions others than NAFLD (2 cirrhosis and 2 hemochromatosis). Ultimately, a total of 44 patients who underwent bariatric surgery were included in the study. Pregnant women and individuals with active neoplasms, recent cardiac instability, severe pulmonary hypertension, or class III or IV heart failure were not included.

### Data collection

All measurements, including anthropometric assessments, blood tests, medical history documentation, and contrast-enhanced ultrasound (CEU) of the carotid artery were conducted during the month prior to the scheduled surgery, except for the liver biopsy, which was performed during the surgery itself.

Height and weight were conducted following established protocols, and body mass index (BMI) was computed as the ratio of weight to the square of height (kg/m²) (19). Blood samples were obtained after an overnight fast, typically between 8:00 and 10:00 AM, from the antecubital vein. Subsequently, the samples were subjected to centrifugation (2000g at 4 °C for 20 minutes), and aliquots were stored at -80°C for later batched analyses. Standard techniques were employed for the determination of biochemical parameter at the clinical biochemistry laboratory within our hospital. Smoking status (never, former, or current smoker) was also obtained. Smokers who quit smoking a year or more before the visit were considered non-smokers.

### Standard and contrast-enhanced carotid ultrasound

CEU was utilized to assess carotid adventitial VV density, employing a Siemens Sequoia 512 ultrasound system equipped with a 15L8W linear array probe and ultrasound contrast software utilizing cadence-contrast pulse sequencing technology. A contrast agent composed of a phospholipid shell encapsulating sulphur hexafluoride was introduced (Sonovue; Bracco Spa, Milan, Italy). Following solubilization in 5 ml of saline, a 2.5ml bolus of the contrast agent was administered via the antecubital vein for each explored carotid artery, using a 20-gauge needle to prevent microbubble rupture.

Quantification of adventitial VV content in the far adventitial layer involved determining the average ratios of intensities above the intima-lumen boundary by 2mm and the intensities below the media-adventitia boundary by 2mm within the common carotid artery, situated 1cm proximal to the bifurcation. The resulting value, denoted as VV signal, was computed as the average of the t10 to t20 ratios derived from diastolic frames characterized by both high and stable lumen and adventitial intensities within a 1-minute video recording (17, 20). VV measurements were performed for both the right and left carotid arteries, and the mean signal for both sides was presented. As a ratio, VV signal lacks units. All CEU studies were digitally stored for subsequent analysis and quantified by an investigator who remained blind to the data.

Additionally, all participants underwent B-mode ultrasound examination of the extracranial carotid arteries to determine the common carotid arteries far wall cIMT, in accordance with previous descriptions (21). We used a Vivid-I ultrasound machine (General Electric Healthcare, Waukesha, WI, USA) coupled with a 12 L-RS linear array transducer probe (5–13 MHz). Measurements of cIMT were taken at 1 cm proximal to the bifurcation, at 1 cm within the bifurcation, and in the initial cm of the internal carotid (22). Segments with atheromatous plaques, denoted by a focal intima-media thickness 1.5 mm extending into the lumen, were excluded from these measurements.

### Liver biopsy

During the bariatric surgery, a wedge liver biopsy was conducted following the established routine surgical protocol. A tissue sample measuring approximately 10x5 mm was obtained from the subcapsular region of the left lobe, specifically segment III as per the Couinaud classification (23). The liver biopsy specimens were fixed in formalin and subsequently embedded in paraffin. An experienced pathologist performed the histopathological evaluation using a semiquantitative approach in accordance with the Clinical Research Network for Nonalcoholic Liver Disease recommendations (24). The reported histopathological findings encompassed the percentage of hepatocytes with both macro and microvesicular steatosis, the identification of Mallory bodies, the presence of fibrosis, as well as the existence of lobular or portal inflammation. The quantification of hepatocytes with steatosis was accomplished through a visual semiquantitative method, with liver steatosis defined as the presence of fatty infiltration in over 5% of hepatocytes. Additionally, the NAFLD activity score (NAS) was determined, along with the presence or absence of nonalcoholic steatohepatitis (NASH). A NAS score of ≥5 and the occurrence of hepatocyte ballooning, on the other hand, identified NASH (24).

### Statistical analysis

The normal distribution of variables was assessed using the Shapiro-Wilk test and evaluating skewness and kurtosis. Continuous variables with a normal distribution were presented as mean values ± SD, while non-normally distributed variables were reported as median and interquartile range (IQR). Categorical variables were presented as percentages. Group differences were compared using the Student t-test for normally distributed data, the Mann-Whitney U test for non-normally distributed data, and χ² for categorical variables. Prior to conducting the Student t-test, the homogeneity of variances was examined using the Levene test. Furthermore, a correlation analysis between VV density and the other variables was conducted by calculating Spearman’s correlation coefficient (rho).

To explore variables independently associated with adventitial VV density, stepwise multivariate regression analyses were employed. The independent variables included in the analyses were age, gender, blood pressure, type 2 diabetes, BMI, serum aspartate aminotransferase, alanine aminotransferase, gamma-glutamyl transferase, glycated hemoglobin, fasting plasma glucose, low density lipoproteins cholesterol, triglycerides, and the presence of simple steatosis or NASH. A separate analysis was conducted to explore the independent variables associated with cIMT. All p-values were derived from two-sided tests of statistical significance, and significance was accepted at the level of P < 0.05. The statistical analyses were carried out using the Stata statistical package (StataC version 16).

## Results

**Table 1** displays the primary clinical characteristics and metabolic data of the study population. Among the participants, thirty-three individuals (75%) exhibited simple steatosis, while eleven were diagnosed with NASH based on liver biopsy findings. Although a greater proportion of males was noted among patients with NASH, no discernible distinctions were observed in terms of age, BMI, and smoking history. Likewise, both groups showed similarities in metabolic parameters encompassing glucose metabolism, lipid metabolism, and liver profile.

**Table 1:** Baseline main clinical and metabolic characteristics of patients in the study.

	Simple steatosis (n=33)	Nonalcoholic steatohepatitis (n=11)	P
**Age (years)**	46.3 ± 11.3	51.1 ± 9.6	0.884
**Woman, n (%)**	30 (90.9)	3 (27.2)	0.034
**BMI (kg/m^2^)**	45.3 ± 6.3	42.3 ± 6.2	0.086
**Non-smoker, n (%)**	10 (30.3)	6 (54.5)	0.975
**Type 2 diabetes, n (%)**	21 (63.6)	7 (63.6)	0.635
**Fasting plasma glucose (mg/dl)**	104 (96 to 116)	114 (95 to 175)	0.349
**HbA1c (%)**	5.7 (5.2 to 6.3)	6.9 (5.7 to 7.9)	0.109
**c-LDL (mg/dl)**	110.4 ± 33.0	104.5 ± 33.8	0.307
**Triglycerides (mg/dl)**	140 (106 to 157)	142 (94 to 223)	0.550
**Hypertension, n (%)**	18 (54.5)	5 (45.4)	0.601
**AST (UI/I)**	21 (18 to 25)	22 (16 to 27)	0.817
**ALT (UI/I)**	27 (17 to 33)	23 (15 to 35)	0.455
**GGT (UI/I)**	29 (17 to 33)	30 (19 to 49)	0.870

Data are mean ± SD, median (interquartile range) or n (percentage). BMI, body mass index; HbA1c, glycated hemoglobin; c-LDL, low-density lipoprotein cholesterol; AST, aspartate aminotransferase; ALT, alanine aminotransferase; GGT, gamma-glutamyl aminotransferase.

Upon evaluating adventitial VV density in both groups, no discernible differences emerged between patients with simple steatosis and NASH, neither in the mean values [0.759 ± 0.104 vs. 0.780 ± 0.043, P=0.702] nor in the left nor right side (**Table 2**). Similarly, no variations were observed when measuring cIMT in both groups.

**Table 2:** Baseline mean adventitial VV density according to steatosis or steatohepatitis condition.

	Simple steatosis (n=33)	Nonalcoholic steatohepatitis (n=11)	P
**VV mean**	0.759 ± 0.104	0.780 ± 0.043	0.702
**VV right**	0.765 (0.693 to 0.850)	0.762 (0.647 to 0.952)	0.654
**VV left**	0.717 (0.639 to 0.783)	0.678 (0.624 to 0.882)	0.935
**cIMT mean (mm)**	0.702 ± 0.140	0.775 ± 0.112	0.934
**cIMT right (mm)**	0.700 ± 0.150	0.769 ± 0.132	0.903
**cIMT left (mm)**	0.718 ± 0.163	0.801 ± 0.147	0.916

Data are mean ± SD or median (interquartile range). VV, vasa vasorum; cIMT, carotid intima-media thickness. Regarding the data from the four patients who were initially excluded due to a diagnosis of healthy liver from the liver biopsy, their mean VV and cIMT were 0.691 ± 0.092 and 0.666 ± 0.171, respectively. There were no significant differences between the groups (healthy liver, simple steatosis, and nonalcoholic steatohepatitis) in the ANOVA analysis (p = 0.471 and p = 0.689, respectively).

**Figure 2:**
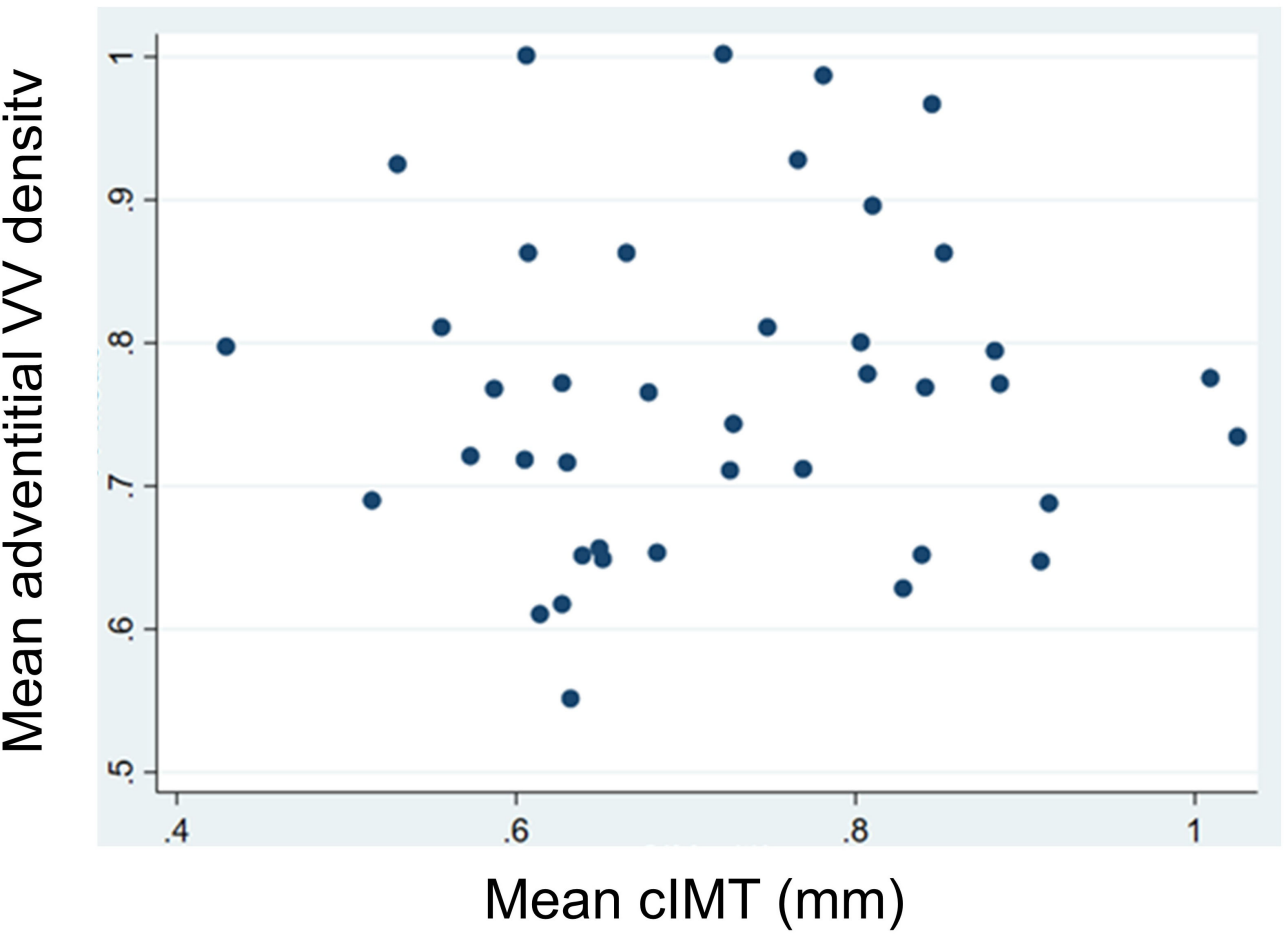
Linear correlations between mean adventitial vasa vasorum (VV) density and mean carotid intima-media thickness (cIMT).

In the univariate analysis, we did not observe any significant correlations between mean adventitial VV and cIMT (r=0.041, P=0.795), BMI (r=-0.017, P=0.908), and systolic blood pressure (r=0.028, P=0.854), nor with the other analytical variables (**Table 3**; **Figure 2**).

**Table 3:** Linear correlations between mean adventitial vasa vasorum density and clinical, anthropometric, and analytical variables in the entire study population.

	r	P-value
**GGT (UI/I)**	-0.193	0.208
**HbA1c (%)**	0.192	0.210
**Triglycerides**	0.153	0.321
**Fasting plasma glucose (mg/dl)**	0.106	0.493
**c-LDL (mg/dl)**	-0.102	0.508
**AST (UI/I)**	-0.076	0.623
**cIMT (mm)**	0.041	0.795
**ALT (UI/I)**	-0.034	0.823
**Systolic blood pressure (mmHg)**	0.028	0.854
**BMI (kg/m^2^)**	-0.017	0.908

GGT, gamma-glutamyl aminotransferase; HbA1c, glycated hemoglobin; c-LDL, low-density lipoprotein cholesterol; AST, aspartate aminotransferase; cIMT, carotid intima-media thickness; ALT, alanine aminotransferase; BMI, body mass index;

In the stepwise multiple linear regression analysis, only gender demonstrated an association with the mean adventitial VV density, while the degree of NAFLD and the other variables did not show significant associations (**Table 4**). Indeed, the mean adventitial VV density was significantly higher in men when compared to women within the entire group (0.856 ± 0.137 vs. 0.747 ± 0.102, P=0.009), as well as in patients with steatosis (0.881 ± 0.096 vs. 0.747 ± 0.784, P=0.016). On the other hand, age and gender but not the severity of NAFLD were found to be the sole variables independently associated with cIMT, with no significant association observed with the mean VV density (**Table 5**).

**Table 4:** Stepwise multiple linear regression analysis of variables associated with the mean adventitial VV density.

Mean adventitial vasa vasorum density		
	Point Estimation (beta)	P-value
**Gender (women)**	-0.206	0.005
**GGT (UI/I)**	-0.001	0.106
**BMI (kg/m^2^)**	0.006	0.154
**Systolic BP (mmHg)**	-0.027	0.154
**c-LDL (mg/dl)**	<0.001	0.195
**Steatosis/Steatohepatitis**	-0.061	0.199
**Age (years)**	0.003	0.255
**HbA1c (%)**	0.042	0.291
**AST (UI/I)**	0.004	0.306
**Triglycerides (mg/dl)**	<0.001	0.347
**Fasting plasma glucose (mg/dl)**	-0.000	0.434
**ALT (UI/I)**	-0.002	0.457

GGT, gamma-glutamyl aminotransferase; BMI, body mass index; BP, blood pressure; c-LDL, low-density lipoprotein cholesterol; HbA1c, glycated hemoglobin; AST, aspartate aminotransferase; ALT, alanine aminotransferase.

**Table 5:** Stepwise multiple linear regression analysis of variables associated with the mean carotid intima-media thickness.

Mean carotid intima-media thickness		
	Point Estimation	P-value
**Age (years)**	0.008	0.001
**Gender (women)**	-0.016	0.033
**c-LDL (mg/dl)**	0.001	0.106
**Fasting plasma glucose (mg/dl)**	-0.001	0.121
**Triglycerides (mg/dl)**	<0.001	0.184
**BMI (kg/m^2^)**	0.004	0.277
**VV mean (mm)**	-0.210	0.280
**HbA1c (%)**	0.041	0.299
**Steatosis/Steatohepatitis**	-0.028	0.556
**AST (UI/I)**	0.001	0.718
**GGT (UI/I)**	<0.001	0.910
**ALT (UI/I)**	<0.001	0.934
**Systolic BP (mmHg)**	-0.003	0.945

c-LDL, low-density lipoprotein cholesterol; BMI, body mass index; VV, vasa vasorum; HbA1c, glycated hemoglobin; AST, aspartate aminotransferase; GGT, gamma-glutamyl aminotransferase; ALT, alanine aminotransferase; BP, blood pressure.

## Discussion

To the best of our knowledge, this study represents the first attempt to evaluate whether NAFLD itself plays a role in the initiation of atherosclerosis, as assessed by adventitial VV density, in patients with severe obesity. Our findings indicate that early stages of atheromatosis are not conditioned by the presence of inflammation and fibrosis within the liver. In our investigation, a comparison of adventitial VV density (mean, right, and left sides) between patients with simple steatosis and those with NASH, characterized by inflammation progressing through fibrosis and structural liver changes, failed to reveal significant differences. Likewise, there was minimal discernible difference in cIMT between patients diagnosed with simple steatosis and NASH.

While CV disease stands as the primary cause of mortality in individuals with NAFLD, consensus remains elusive regarding whether NAFLD confers increased cardiovascular risk independently of established risk factors (10, 12). The co-occurrence of NAFLD and these CV risk factors complicates epidemiological studies seeking to discern whether liver changes contribute to CV disease. In line with this, a 2020 expert consensus has proposed replacing the term NAFLD with a more accurate descriptor: Metabolic Associated Fatty Liver Disease (MAFLD) (25, 26). Targher et al., through a meta-analysis encompassing 16 studies and 34,043 subjects, found NAFLD to correlate with fatal and non-fatal cardiac events, a relationship that intensified with NAFLD severity (27). Conversely, Wu et al. reported, in a meta-analysis of 34 studies and 164,949 subjects, significant associations between NAFLD and increased prevalence of atherosclerosis and CV disease, while failing to establish significant NAFLD-CV disease mortality or overall mortality links (28). In concurrence, a more recent meta-analysis by Liu et al. involving 24 studies and 498,501 subjects demonstrated elevated all-cause mortality risk in NAFLD patients (HR = 1.34, 95% CI 1.17-1.54), but again failed to establish a significant link between NAFLD and CVD mortality (29). In our study, we did not observe any differences in the prevalence of CV risk factors, such as type 2 diabetes, hypertension, or dyslipidemia, between patients with simple steatosis and NASH. This reinforces the idea that the severity of NAFLD does not have an impact in the initial phases of atheromatous disease.

Prior studies have explored the relationship between NAFLD and surrogate markers of CV disease and endothelial dysfunction, such as cIMT, aortic stiffness, brachial artery vasodilatory responsiveness, and coronary artery calcification (30–36). However, our study is unique in its assessment of early morphofunctional changes in the arterial wall based on NAFLD stage, showing that NASH is not associated with increased VV density. In advanced stages of atherosclerotic disease, a study involving 4,222 individuals in Germany revealed a notable increase in carotid atherosclerotic plaque prevalence among those diagnosed with hepatic steatosis through ultrasound (30). However, even after accounting for CV risk factors, no significant differences in cIMT were observed (30). Additionally, Styczycnski et al.’s study of 120 patients with severe obesity who underwent bariatric surgery found no association between aortic stiffness and NAFLD severity defined by wedge liver biopsy (36). Conversely, other studies have yielded affirmative findings in the association between steatosis and atherosclerotic disease. For example, a 5 years longitudinal study including 728 men and 497 women free of hypertension and type 2 diabetes at the baseline noted accelerated arterial stiffness progression in NAFLD individuals, regardless of metabolic syndrome presence (35). Similarly, in 52 NAFLD cases and 28 age- and sex-matched controls, NAFLD correlated with reduced brachial artery vasodilatory response to ischemia (31). Two Korean studies involving 1,854 and 10,153 individuals, respectively, reported elevated coronary artery calcification scores in NAFLD patients (32, 33). While our approach aligns with the gold standard method for diagnosing non-alcoholic fatty liver disease, most of these studies relied on ultrasound-based NAFLD diagnoses, potentially skewing participant inclusion toward advanced steatosis and metabolic abnormalities (30, 32–35).

Our study also demonstrated the absence of a linear correlation between adventitial VV density and cIMT in patients with severe obesity, suggesting that we may be assessing different stages in the development of atheromatous disease, with different etiopathogenic factors and clinical situations responsible for their stimulation. This data further supports the notion that NAFLD exerts limited influence on the early phases of atheromatous disease when evaluated in two different ways. Insights into the absence of association between NAFLD and CV disease association may also emerge from naturally occurring mutations in genes affecting liver fat content (11, 37, 38). Recent evidence highlights genetic variants predisposing to NAFLD that lack a corresponding elevated CV disease risk in the absence of metabolic syndrome components (39, 40). Supporting this, a mendelian randomization study of two large European cohorts found no causal link between NAFLD and CV disease (40).

Surprisingly, modifiable factors such as blood pressure, type 2 diabetes, and lipid profile did not emerge as independent predictors of VV density, with only gender demonstrating such influence. Therefore, our results underscore gender’s role in atherosclerotic disease, influencing etiology, clinical presentation, and prognosis of patients with cardiovascular disease (41–43). Evidence suggests that atheromatous disease manifests 10 to 15 years earlier in men than women, particularly during reproductive age. Postmenopausal women face increased CV disease risk due to estrogen secretion cessation, leading to comparable incidence rates between older women and men (42, 44). In our relatively younger study population with severe obesity, over half of whom exhibited type 2 diabetes or hypertension, gender may contribute to VV density differences. These results align with previous research by Akabame et al. and Wong et al., which indicated a gender imbalance in CV disease rates (65% male vs. 35% female; 70.8% male vs. 29.2% female, respectively) (45, 46). Moreover, our study suggests that male gender’s negative impact may extend to initial CV disease stages, as indicated by an independent association with increased VV density and increased cIMT in multiple regression analysis.

Several limitations merit discussion regarding our study. Primarily, its cross-sectional design precludes both causality and longitudinal follow-up. Additionally, data collection from a single hospital potentially limits representation of the broader fatty liver disease population. Notably, VV density and cIMT were measured solely in NAFLD patients, precluding a comparison to histologically normal liver individuals. However, measurements from 141 overweight men and women without established coronary heart disease or a risk equivalent, such as diabetes, from a multiracial and multiethnic population in the metropolitan area of Chicago, Illinois, showed results similar to those described in our study for both for the average median adventitial VV ratio (0.80 ± 0.19) and cIMT (0.82 ± 0.22) (47). Second, our study’s small sample size, including 11 NASH patients and 33 with simple steatosis, constitutes a further limitation and precludes to generalize our results to the entire population. However, both groups exhibited comparable clinical and biochemical profiles except for gender, bolstering our findings. Of significant note, our study utilized incidental liver biopsies conducted during scheduled bariatric procedures to diagnose cases of simple steatosis and steatohepatitis. While this approach aligns with the gold standard method for diagnosing NAFLD, it is worth mentioning that certain other liver biopsy studies employed a selection criterion of elevated transaminase levels, suggestive of the presence of NASH and more advanced metabolic abnormalities. Finally, previous studies have demonstrated the negative impact of BMI on VV density and cIMT (48). In our study, although BMI does not appear as a variable independently associated with adventitial VV density, the lack of BMI variability among the study population, which consists of individuals with severe obesity who underwent bariatric surgery, should be kept in mind for future studies.

In summary, our findings suggest that NASH does not contribute to increased VV density or cIMT compared to simple steatosis. This study emphasizes the complex interplay between NAFLD and CV disease, highlighting the importance of considering other risk factors when evaluating cardiovascular risk in NAFLD patients. Further research is needed to understand the nuanced relationships between NAFLD, atherosclerosis, and gender in larger, more diverse populations.

## Data availability statement

The raw data supporting the conclusions of this article will be made available by the authors, without undue reservation.

## Ethics statement

The studies involving humans were approved by Human Ethics Committee at Arnau de Vilanova University Hospital (CEIC-1275). The studies were conducted in accordance with the local legislation and institutional requirements. The participants provided their written informed consent to participate in this study.

## Author contributions

JL-M: Conceptualization, Formal analysis, Investigation, Software, Writing – original draft, Writing – review & editing. ES: Conceptualization, Investigation, Writing – review & editing. FH: Investigation, Writing – review & editing. MC: Investigation, Writing – review & editing. MS: Investigation, Writing – review & editing. JV: Methodology, Writing – review & editing. MB-L: Data curation, Methodology, Writing – review & editing. EC: Conceptualization, Investigation, Writing – review & editing. JP: Investigation, Writing – review & editing. XM-G: Investigation, Writing – review & editing. FV: Investigation, Writing – review & editing. AC: Supervision, Writing – review & editing. MBu: Data curation, Writing – review & editing. RM: Data curation, Writing – review & editing. AL: Conceptualization, Formal analysis, Investigation, Methodology, Project administration, Software, Supervision, Validation, Writing – review & editing.
